# Virtual contrast-enhanced maximum intensity projections from high-*b*-value diffusion-weighted breast MRI: a feasibility study

**DOI:** 10.1186/s41747-025-00625-7

**Published:** 2025-10-08

**Authors:** Andrzej Liebert, Hannes Schreiter, Dominique Hadler, Lorenz A. Kapsner, Sabine Ohlmeyer, Jessica Eberle, Ramona Erber, Julius Emons, Frederik B. Laun, Michael Uder, Evelyn Wenkel, Sebastian Bickelhaupt

**Affiliations:** 1 Institute of Radiology, Universitätsklinikum Erlangen, Friedrich-Alexander-Universität Erlangen-Nürnberg (FAU), Erlangen, Germany; 2 Medical Center for Information and Communication Technology, Universitätsklinikum Erlangen, Friedrich-Alexander-Universität Erlangen-Nürnberg (FAU), Erlangen, Germany; 3 Institute of Pathology, Universitätsklinikum Erlangen, Erlangen, Comprehensive Cancer Center Erlangen-EMN, Friedrich-Alexander-Universität Erlangen-Nürnberg (FAU), Erlangen, Germany; 4 Institute of Pathology, University Regensburg, Regensburg, Germany; 5 Department of Gynecology and Obstetrics, Erlangen University Hospital, Comprehensive Cancer Center Erlangen-EMN, Friedrich-Alexander-Universität Erlangen-Nürnberg (FAU), Erlangen, Germany; 6 Medizinische Fakultät, Friedrich-Alexander-Universität Erlangen-Nürnberg (FAU), Erlangen, Radiologie München, München, Germany; 7 German Cancer Research Center (DKFZ), Heidelberg, Germany

**Keywords:** Artificial intelligence, Breast, Multiparametric magnetic resonance imaging, Neoplasms, Neural networks (computer)

## Abstract

**Background:**

Maximum intensity projections (MIPs) facilitate rapid lesion detection both for contrast-enhanced (CE) and diffusion-weighted imaging (DWI) breast magnetic resonance imaging (MRI). We evaluated the feasibility of AI-based virtual CE subtraction MIPs as a reading approach.

**Materials and methods:**

This Institutional Review Board-approved retrospective study includes 540 multi-parametric breast MRI examinations (performed from 2017 to 2020), including multi-*b*-value DWI (50, 750, and 1,500 s/mm²). A 2D U-Net was trained using unenhanced (UnE) images as inputs to generate virtual abbreviated CE (VAbCE) subtractions. Two radiologists evaluated lesion suspicion, image quality, and artifacts for UnE, VACE, and abbreviated CE (AbCE) images. Lesion conspicuity was compared between VAbCE and AbCE MIPs.

**Results:**

Cancer detection rates for UE, VAbCE, and AbCE MIPs were 90.0%, 91.4%, and 94.3%, respectively. Single-slice reading demonstrated sensitivities of 88.6% (UnE), 91.4% (VAbCE), and 94.3% (AbCE). Inter-rater agreement (Cohen κ) for lesion suspicion scores was higher for VAbCE (0.53) than UnE alone (0.39) and comparable to AbCE (0.58). No significant difference in mean lesion conspicuity was observed for VACE MIPs compared to ACE (*p* ≥ 0.670). No significant difference could be observed for quality (*p* ≥ 0.108), and reading time (*p* = 1.000) between methods. Fewer visually significant artifacts could be observed in VAbCE than in AbCE MIPs (*p* ≤ 0.001).

**Conclusion:**

VAbCE breast MRI improved inter-rater agreement and allowed for slightly improved sensitivity compared to UnE images, while AbCE still provided the overall highest sensitivity. Further research is necessary to investigate the diagnostic potential of VAbCE breast MRI.

**Relevance statement:**

VAbCE breast MRI generated by neural networks allowed the derivation of MIPs for rapid visual assessment, showing a way for screening applications.

**Key Points:**

- Virtual abbreviated contrast-enhanced (VAbCE) MIPs provided comparable sensitivity to MIPs of unenhanced high *b*-value DWI and were slightly lower than AbCE MIPs.
- Adding VAbCE to unenhanced high *b*-value DWI significantly improved interrater agreement for lesion suspicion scoring.
- Single-slice evaluation of VAbCE MIPs provided a sensitivity comparable to unenhanced high *b*-value DWI MIPs.

**Graphical Abstract:**

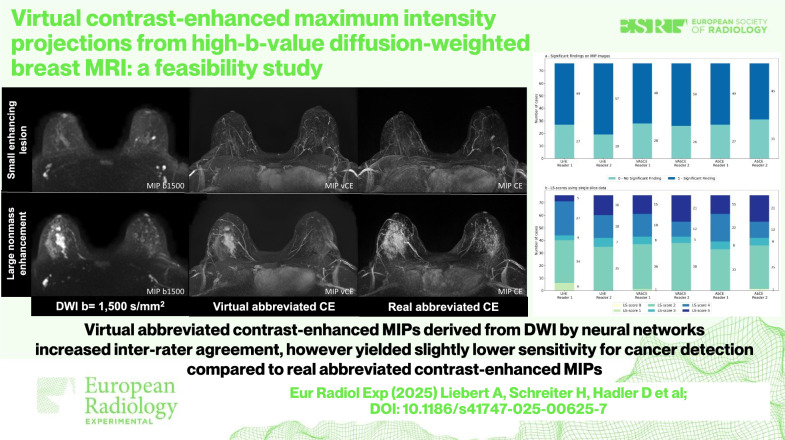

## Background

Breast MRI is increasingly investigated with regard to its capability to supplement early detection of breast cancer [1–4]. Breast MRI protocols commonly include a multiparametric acquisition protocol, consisting of morphologic T1-weighted and T2-weighted as well as diffusion-weighted imaging (DWI) sequences and the pivotal contrast-enhanced (CE) sequences acquired after the administration of gadolinium-based contrast agents [5, 6].

Reading of CE breast MRI routinely includes subtraction series, which are derived from sequences before and after contrast administration, highlighting tissue enhancement characteristics. Further, such subtraction series allow creating maximum intensity projections (MIPs), which summarize the maximum intensity from the entire stack of slices of the acquisition volume [7]. Assessment of the resulting two-dimensional MIPs has been suggested for the identification of potentially relevant breast MRI findings subsequently needing further evaluation on individual slices [7]. MIPs might be of special value when it comes to introducing breast MRI to a high-throughput scenario such as breast cancer screening. Diagnostic performance assessments of subtraction MIPs from abbreviated CE (AbCE) protocols suggest a fast and reliable lesion detection approach for screening [7, 8].

Research interest has emerged toward investigating unenhanced (UnE) breast MRI, which is typically performed with the acquisition of DWI and T2-weighted fat-saturated sequences. Such a combination of sequences allows for evaluation of tissue composition and fluid presence in high resolution (through the latter sequence) and, at the same time, to identify suspicious regions of decreased water molecule diffusivity, which are hallmarks of malignant lesions (through the former sequences). The UnE abbreviated protocols have the potential to further improve accessibility to breast MRI by reducing the periprocedural time/costs related to gadolinium-based contrast agent administration [9, 10]. While limited in spatial resolution as compared to T1-weighted and T2-weighted sequences, DWI, including high *b*-value acquisitions, has recently been suggested to allow even for morphologic lesion characterization [11]. Using artificial intelligence to create virtual CE images has been discussed as a potential mediator, mitigating limitations of UnE breast MRI by providing a visual image mimicking the characteristics of CE sequences [12–23].

In this feasibility study, we evaluated MIPs and the subsequent reading of the individual single-slice data of three abbreviated MRI approaches: (a) UnE MRI with high *b*-value DWI; (b) UnE MRI augmented by artificial intelligence in order to derive images mimicking the characteristics of CE subtraction MIPs; and (c) the original CE subtraction data. To our knowledge, this is the first study on MIPs from virtual CE breast MRI.

## Materials and methods

### Study cohort

This retrospective study was approved by the ethics committee of the Medical Faculty of Friedrich-Alexander Universitat Erlangen-Nurnberg, waiving the need for informed consent.

The study included 540 female patients, aged 52 ± 12 years (mean ± standard deviation), with breast MRI examination acquired between 2017 and 2020 at the Erlangen University Hospital. The inclusion criteria were:

1. clinical indication for breast MRI, *e.g*., preoperative exclusion of multifocal disease, screening in women with positive family history for breast cancer, exclusion of recurrent breast cancer, clarification of unclear findings in mammography/ultrasound or clinical complaints;
2. examinations performed on the most commonly used scanner type at University Hospital Erlangen during the chosen study period (3-T Siemens MAGNETOM Skyra Fit, Siemens Healthineers, Erlangen, Germany);
3. examinations performed with a full multiparametric diagnostic protocol, including DWI with three different *b*-values (50, 750, 1,500 s/mm^2^), T2-weighted fat-saturated sequence, and a T1-weighted dynamic GBCA-enhanced (DCE) series.

The dataset was randomly divided, without any specific stratification in regard to the clinical parameters, into a training and validation set with 70% (*n* = 377) and 15% (*n* = 81), respectively, while the remaining 15% (*n* = 82) of the whole dataset remained untouched as an independent test, which was used for the reader study. After the performance of the data readings, three patients had to be excluded from the test set due to missing histopathological information, and three patients had to be excluded due to undergoing neoadjuvant chemotherapy without a visible residual finding in the original diagnostic report. Single board-certified radiologist (S.B., over 10 years of experience) evaluated the breast tissue characteristics and background-parenchymal enhancement based on the Breast Imaging reporting and Data System (BI-RADS) evaluation scheme [24].

The summary of the patient cohort with a split into the training, validation, and independent test set is presented in Table 1.

**Table 1:** Patient demographics

	Whole dataset	Training	Validation	Test
Number of patients	540 (534)***	377	81	82 (76***)
Number of exclusions	6***	0	0	6***
Split ratio (%)		70%	15%	15%
Age (years)*	52 ± 12	52 ± 12	50 ± 11	52 ± 13
Patients with significant findings (BI-RADS ≥ 3)**	306 (57.3%)	223 (59.2%)	47 (58.0%)	35 (46.1%)
Examinations with malignant findings	231 (43.3%)	167 (44.3%)	29 (35.8%)	35 (46.1%)
Breast tissue type				
Almost entirely fat	50 (9.4%)	38 (10.1%)	7 (8.6%)	5 (6.6%)
Scattered	190 (35.6%)	142 (37.8%)	24 (29.6%)	24 (31.6%)
Heterogeneously dense	203 (38.1%)	138 (36.7%)	31 (38.3%)	34 (44.7%)
Extremely dense	90 (16.9%)	58 (15.4%)	19 (23.5%)	13 (17.1%)
Background parenchymal enhancement				
Minimal	48 (9.0%)	28 (7.4%)	11 (13.6%)	9 (11.8%)
Mild	237 (44.5%)	174 (46.3%)	28 (34.6%)	35 (46.1%)
Moderate	183 (34.1%)	129 (34.3%)	31 (38.3%)	22 (28.9%)
Marked	66 (12.4%)	45 (12.0%)	11 (13.6%)	10 (13.2%)

* Age is given as mean ± standard deviation

** Significant findings were defined as BI-RADS ≥ 3 in the radiologist’s report in the clinical routine reading

*** Final number of analyzed patients, since three patients were excluded due to a lack of clinical/histopathological reference data and three patients due to having a histopathologically confirmed cancer but undergoing neoadjuvant chemotherapy, with the radiologist’s report stating no residual finding

### MRI

All acquisitions were performed in prone position using a 3-T MRI (Skyra Fit, Siemens Healthineers) with an 18-Channel breast coil (Siemens Healthineers). Detailed information about the acquisition parameters of the multiparametric full-diagnostic-protocol is presented in Table 2.

**Table 2:** Sequence parameters of the MRI sequences used in the study

Sequence	Matrix size × number of slices	Field of view (mm × mm)	Slice thickness (mm)	TR (ms)	TE (ms)	IR (ms)	Number of averages	Additional parameters
T1-weighted	448 × 448 × 112–128	360 × 360–430 × 430	1.5–1.8	5.97	2.46	–	1	Acquired before and at 5 time points after contrast injection
T2-weighted	448 × 448 × 34–49	340 × 340–430 × 430	4	3,570–5,020	60, 70	230	2	Fat-saturation
DWI	256 × 160–200 × 34–49	350 × 219–430 × 269	4	6,290–9,660	66, 70	220, 250	3 (*b*-value 50 s/mm^2^) 8 (*b*-value 750 s/mm^2^) 20 (*b*-value 1,500 s/mm^2^)	Fat-saturation, three *b*-values: 50,750, 1,500 s/mm^2^

*DWI* Diffusion-weighted imaging*, IR* Inversion recovery time, *TE* Echo time, *TR* Repetition time

### Histopathology

Diagnostic ground truth was established for the independent test set as previously described by Ohlmeyer et al [25] by evaluating histopathology reports from ultrasound-guided or stereotactic biopsy. For all BI-RADS > 3 lesions in the final test dataset, histopathology served as reference. Cases with BI-RADS 3 were followed up either with a subsequent histopathology verification or deemed benign based on the follow-up examination. Follow-up examinations commonly covered at least 12 months of time. All enhancing lesions longest axis was measured at the inner border by a single reader (research assistant J.E. with over 3 year of experience in breast MRI) under the supervision of a board-certified radiologist (D.H. with over 10 years of experience) in the subtraction images of the 2nd time-point of the T1-weighted DCE series using the open-source software 3D-Slicer (3D Slicer, Version 4.11 [26]). The whole cohort (*n* = 540) was previously included in the evaluation of algorithmic detection or forecasting of image artifacts [27–29], as well as in a study in which the technical feasibility of either generation of virtual DCE or virtual T2-weighted fat-saturated sequences was investigated [22, 30].

### Neural network

For the generation of the virtual contrast-enhanced subtraction sequence, a multichannel 2D-U-net network similar to the architecture presented by Schreiter et al [22] was implemented. During the training of the network, UnE T1-weighted, T2-weighted, and DWI acquisitions with three *b*-values were provided as input data and DCE data were provided as the training targets. The resulting trained network was thus able to generate virtual CE images out of the UnE input data. Details of the neural network architecture, data preprocessing and the training hyperparameters are presented in the Supplemental material.

### Image processing to derive MIPs

In order to perform the reading study, MIPs were calculated. MIPs were derived from: (a) the CE subtraction images from the 2nd time-point of the T1-weighted DCE series; (b) the virtual CE subtraction series generated by the neural network based on the UnE acquisitions; and (c) the high *b*-value DWI acquisitions. In short, for all the above-mentioned series, the maximum intensity of each matrix and slice was registered along the head-feet axis of the entire stack and was projected into a single image. This step was performed using an in-house developed Python script (version 3.8.10, Python Software Foundation).

### Image assessment

Image series including the MIPs were transferred to the research workstation and analyzed using Synedra View 20 Software (Version 20.0008, Synedra IT GmbH). Three abbreviated breast MRI reading schemes were assessed with at least 2 weeks in between the readings being performed by two experienced radiologists (Reader 1 (R1): S.B., over 10 years of experience; Reader 2 (R2): D.H., over 15 years of experience) in the two experiments described below.

### Comparison of abbreviated protocols in simulated reading (experiment 1)

**Fig. 1:**
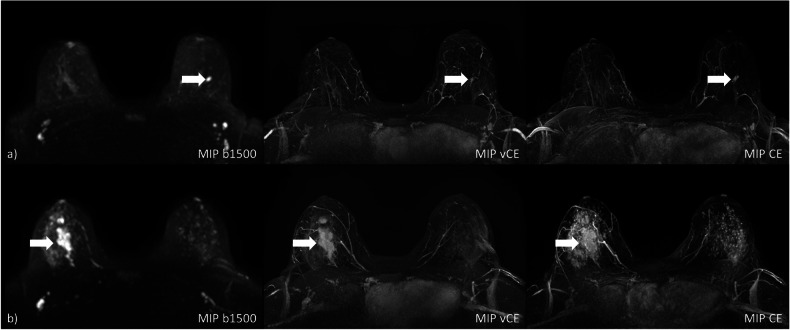
**a** A small GBCA-enhancing lesion is visible in the CE-MIP in the left breast, which can be well identified in both the vCE and *b*-1,500 MIPs (arrows). **b** Large non-mass enhancement in the right breast (arrows), histopathologically confirmed as extensive ductal carcinoma *in situ* (DCIS) in a woman with elevated background parenchymal enhancement in the CE-MIP with both the vCE and *b*-1,500 MIPs, allowing for delineation of the finding with high confidence. *b*-1,500, Diffusion-weighted imaging with *b* = 1,500 s/mm^2^; CE, Contrast-enhanced; GBCA, Gadolinium-based contrast agent; MIP, Maximum intensity projection; vCE, Virtual contrast-enhanced

**Fig. 2:**
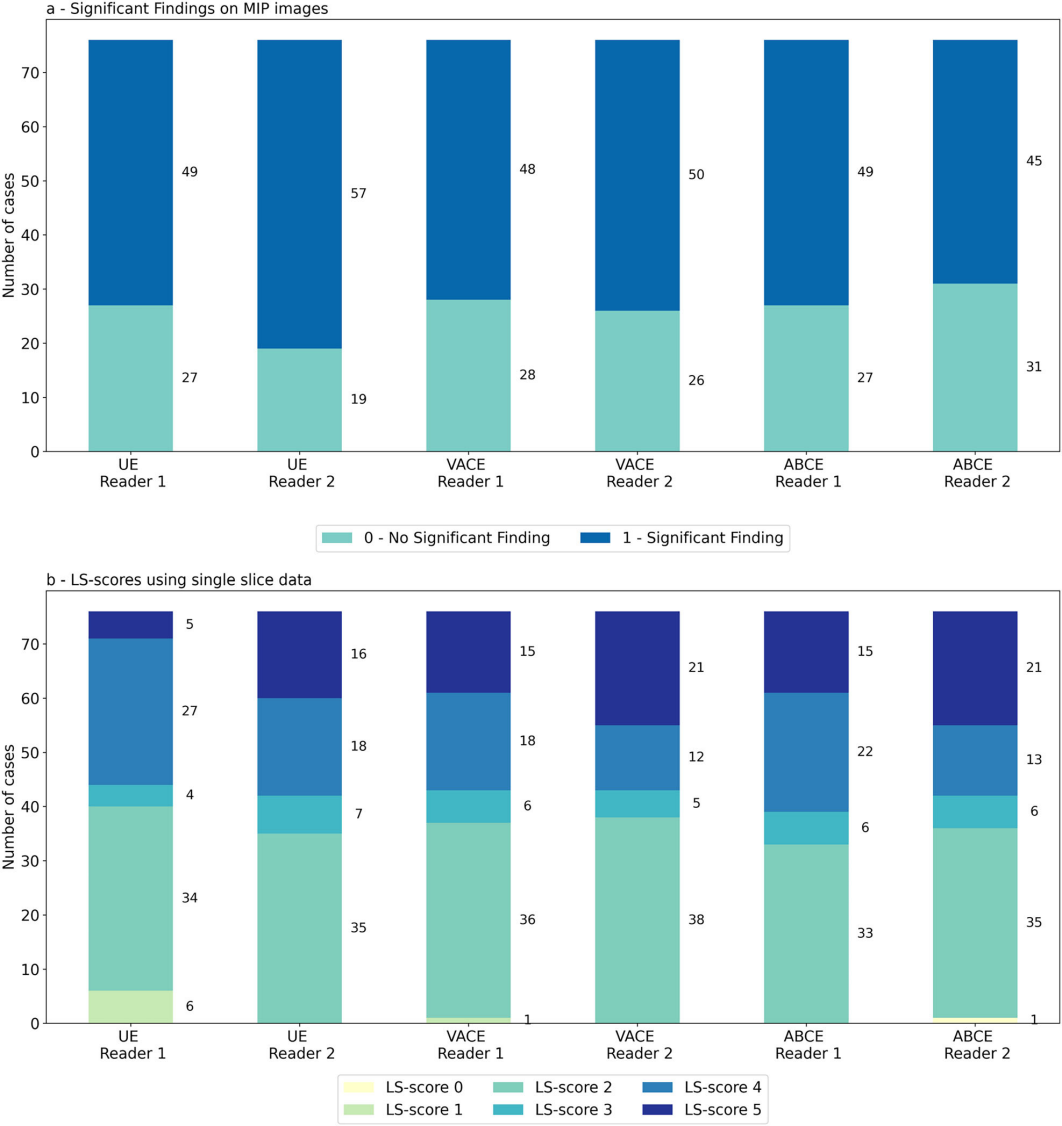
Distribution of significant finding calls (**a**) on MIPs and LS-scores (**b**) evaluated on single-slice images provided by the two readers for the three reading protocols. LS-score, Lesion suspicion score; UnE, Unenhanced; VAbCE, Virtual abbreviated contrast-enhanced; AbCE, Abbreviated contrast-enhanced

**Fig. 3:**
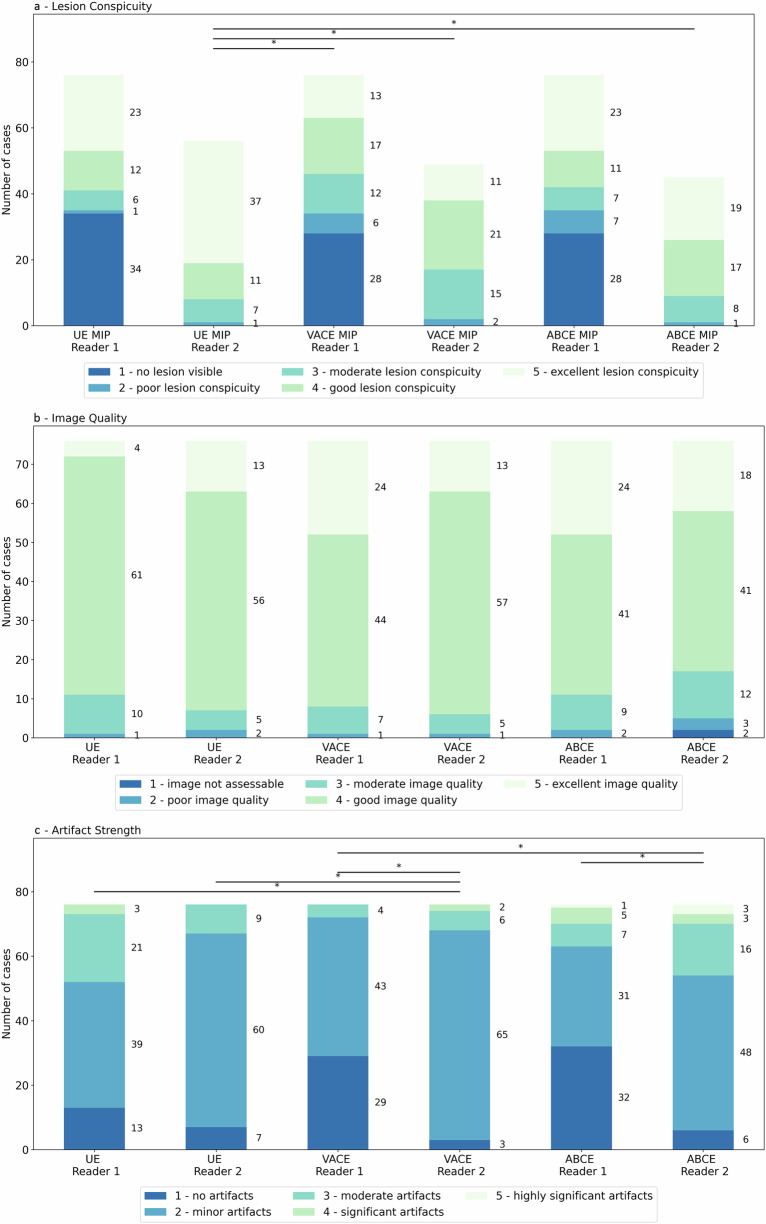
Lesion conspicuity (**a**), image quality (**b**), and artifact evaluation (**c**) on the MIPs for the three methods (UnE, VAbCE and AbCE) for the two readers. Values next to the bars indicate the total number of cases with the respective score. Significant differences in medians between respective readings are marked with stars, indicating a *p*-value < 0.05. UnE, Unenhanced; VAbCE, Virtual abbreviated contrast-enhanced MRI; AbCE, Abbreviated contrast-enhanced

**Fig. 4:**
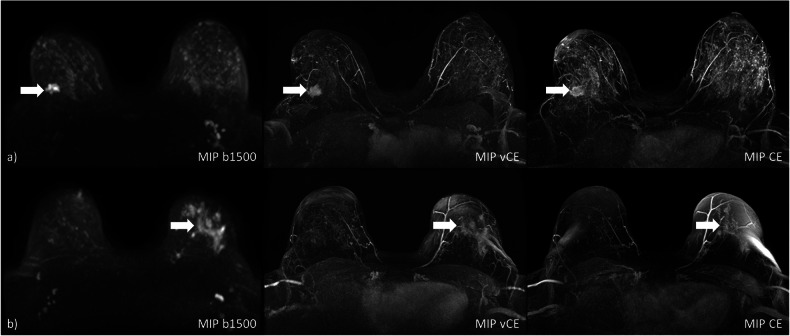
**a** Women with high BPE at CE-MIP with lesion depiction slightly impeded. Both the *b*-1,500- and the vCE-MIP allow a similar lesion delineation (histopathology: invasive carcinoma) (arrows). **b** The vCE-MIP shows a minor artifact potentially interfering with image analysis of the enhacing malignant findings in the left breast, with the artifact being less pronounced compared to the CE-MIP, and the *b*-1,500 MIP shows no significant artifacts at all;. *b*-1,500, Diffusion-weighted imaging with *b* = 1,500 s/mm^2^; CE, Contrast-enhanced; MIP, Maximum intensity projection; vCE, Virtual contrast-enhanced

The first experiment used reading schemes adapted from the basic principles described by Kuhl et al [7] and Ohlmeyer et al [25]. The reader was blinded to all clinical data, imaging reports and previous examinations of the patients. The reader could not be technically blinded to the aspect whether analyzing a CE or virtual CE image since image characteristics slightly but obviously differed (see figures that are presented within the result section, especially Figs. 1–4). The following three abbreviated reading protocols were consecutively evaluated.

#### Unenhanced protocol

In adaptation to previous works on abbreviated UnE breast MRI [25], the first protocol consisted of: (a) T2-weighted fat-saturated sequence; (b) a multi-*b*-value DWI acquisition with *b*-values of 50, 750, 1,500 s/mm^2^; and (c) the MIP derived from the DWI acquisition using the *b*-value of 1,500 s/mm^2^. This protocol is further called the *UnE protocol.*

#### Virtual abbreviated CE protocol

The second protocol consisted of: (a) the virtual CE MRI subtraction series (corresponding to an image contrast replicating the subtraction series of the 2nd time-point after contrast administration, with this time-point internally evaluated to provide the optimal lesion visibility); (b) the MIP derived from this virtual subtraction series; and (c, d, e) the source acquisitions fed to the algorithm, namely the UnE T1-weighted, T2-weighed fat-saturated sequence, and the multi-*b*-value DWI. This protocol is further called the VAvCE protocol.

#### Abbreviated CE protocol

The third protocol consisted of: (a) the original CE subtraction series of the 2nd dynamic acquisition; (b, c) the underlying source images of UnE and CE T1-weighted sequence; and (d) the MIP derived from the CE subtraction series. The 2nd time-point of the dynamic acquisition was chosen with regard to the in-house experience of providing the optimal contrast for the detection of suspicious lesions. This protocol is further called the AbCE protocol.

As the first reading session of each protocol, MIPs were used to initially assess the presence of a visually significant finding. A visually significant finding was defined as the presence of a focal signal enhancement or signal intensity increase beyond the background signal of the tissue [7]. The reading time to perform this step of the analysis was registered using a stopwatch. Further, lesion conspicuity, image quality, and artifact strength of the MIPs were assessed using the following three 5-point Likert scales:

1. lesion conspicuity: 1 = no lesion visible, 2 = poor lesion conspicuity, 3 = moderate lesion conspicuity quality, 4 = good lesion conspicuity, 5 = excellent lesion conspicuity;
2. image quality: 1 = image not assessable, 2 = poor image quality, 3 moderate image quality, 4 = good quality, 5 = excellent image quality;
3. artifacts: 1 = no artifacts at all, 2 = minor artifacts, not impeding the assessment, 3 = moderate artifacts, potentially interfering with the assessment, 4 = visually significant artifacts impeding the assessment, 5 = visually highly significant artifacts, making a reliable assessment impossible; additionally, an investigation of visually significant artifacts was performed in which artifacts of strength > 2 were defined as visually significant artifacts.

In the sequentially following reading session conducted at 2 weeks after each other, complementary independent reading of the full abbreviated protocols for UnE, VAbCE, and the AbCE protocols, allowing for the assessment of all individual slice images of the respective protocol as well as the corresponding MIPs and the respective protocol source images was performed. During each reading session, first the MIP images were evaluated, and then all were followed up by the single-slice reading. In similarity to previous works, between the reading sessions of the respective protocols, the readers had at least a 2-week-long washout period [13, 14, 20].

The readers provided a “BI-RADS adapted” lesion suspicion scoring from 1-to-5 for each protocol, called “LS-score” [25]. The LS-score was categorized in order to match the respective categories described for the BI-RADS scores. For data analysis purposes commonly, the highest LS-score per patient was considered. A classification of findings as LS-score 3 (checkup required) was aimed to be minimized in the evaluation of this study, to better reflect the discouragement for this category in the screening context.

### Side-by-side comparison of image equivalence and individual lesion conspicuity (experiment 2)

In order to directly compare the MIP images of the virtual CE data and the original CE enhanced data, a further experiment was conducted. In an unblinded side-by-side reading, an evaluation adapted from Mueller-Franzes et al [14] was performed by both readers from Experiment 1 to answer, using a 5-point Likert-like scale from 1-to-5, the question if the virtual CE data has an overall matching image appearance as compared to the original CE data (1 = entirely different, 2 = slightly equivalent, 3 = moderately equivalent, 4 = very equivalent, 5 = exactly equivalent) and whether the conspicuity of enhancing lesions is identical in the two datasets (1 = lesion not visible at all to 5 = lesion equivalently enhancing).

### Statistical analysis

The diagnostic performance of the three reading protocols was compared independently for the MIP reading and the analysis of the fully abbreviated protocols by calculating sensitivity, specificity and accuracy. Differences in the diagnostic performance were evaluated using the exact McNemar test, assuming LS-scores ≥ 3 to denote clinically significant findings. Differences in ordered variables between the methods for each of the readers and between the readers were assessed using a Friedman test followed by a Tukey post hoc analysis. All statistical analyses were performed using Sigmaplot software (v15.0, Grafiti LLC) and considered a *p*-value of 0.05 to be significant. For results with significant statistical differences, effect size evaluation was performed using Cliff’s δ for all ordinal scores with more than two classes and odds ratio (OR) for the visually significant artifacts. For comparison of inter-rater agreement between the two readers, Cohen κ was calculated with confidence intervals (CI) evaluation using the bootstrapping method with *n* = 1,000 samples. The inter-rater agreement comparison was performed using Python and the Sci-kit learn library (v1.5.0). Since the investigation of the lesion conspicuity might be affected by artifacts present in the MIP, the correlation between the artifact strength and lesion conspicuity score was investigated using the Spearman correlation coefficient (ρ).

## Results

### Patient demographics in the independent test dataset

Among the independent test cohort (*n* = 76), 35 women, aged 54 ± 11 years (mean ± standard deviation), were diagnosed with a malignant lesion, and 41, aged 50 ± 14 years, presented with benign findings only. The radiological median size of malignant findings in the study group was 22.9 mm (interquartile range 17.8 mm). Benign findings consisted of 14 cysts, 5 fibroadenomas, 2 lymph nodes, 1 sclerosing adenosis, 1 compacted fibro-glandular tissue, 1 seroma, and 17 nonspecific findings. Regarding background parenchymal enhancement, of 76 cases, the minimal class was observed in 17 (22.4%), while mild, moderate and marked classes were observed in 29 (38.2%), 24 (31.5%), and 6 (7.9%), respectively. Regarding the tissue type, 5 (6.6%) were almost entirely fatty; scattered, heterogeneously dense, and extremely dense types were observed in 24 (31.6%), 34 (44.7%), and 13 cases (17.1%), respectively. The demographics data for the training and validation set are provided in Table 1.

### Comparison of abbreviated protocols in simulated reading (experiment 1)

#### Significant findings on MIPs

Figure 1 shows two example cases of the MIPs generated for each of the protocols. Figure 2a shows the distribution of significant finding calls on the MIP images for the two reader and the three protocols. In this initial assessment, the readers achieved a moderate inter-rater agreement for all three methods (UnE, κ = 0.63, CI: 0.44–0.80; VAbCE, κ = 0.71, CI: 0.54–0.86; AbCE, κ = 0.78, CI: 0.62–0.91).

The readers reached a mean cancer detection rate of 90.0% (R1: 85.7%; R2: 94.3%) for the UnE protocol, 91.4% (R1: 88.6%; R2: 94.3%) for the VAbCE protocol, and 94.3% (R1: 94.3%; R2: 94.3%) for the AbCE-derived MIPs. The MIPs achieved a mean specificity of 47.6% (R1: 53.7%; R2: 41.5%), 58.5% (R1: 58.5%; R2: 58.5%) and 65.9% (R1: 61.0%; R2: 70.7%) for the UnE, VAbCE, and AbCE protocols, respectively. Mean accuracies of 67.1% (R1: 68.4%; R2: 65.8%), 73.7% (R1: 72.4%; R2: 75.0%) and 78.9% (R1: 76.3%; R2: 81.6%) could be observed for UnE-, VAbCE- and AbCE-derived MIPs. No significant differences in the accuracy could be observed for R1 (*p* ≥ 0.210) between any of the methods. For R2, a significant difference in the accuracy was observed between the UnE- and the ABCE-MIPs (*p* = 0.004). Detailed investigation of the performance metrics is presented in Table 3.

**Table 3:** Comparison of the diagnostic performance of reading protocols

	Unenhanced abbreviated				Virtual abbreviated CE				Abbreviated CE			
	MIP only		Single-slice reading		MIP only		Single-slice reading		MIP only		Single-slice reading	
	R1	R2	R1	R2	R1	R2	R1	R2	R1	R2	R1	R2
Cancer detection rate	85.7% (30/35)	94.3% (33/35)	82.9% (29/35)	94.3% (33/35)	88.6% (31/35)	94.3% (33/35)	91.4% (32/35)	91.4% (32/35)	94.3% (33/35)	94.3% (33/35)	97.1% (34/35)	91.4% (32/35)
Specificity	53.7% (22/41)	41.5% (17/41)	82.9% (34/41)	80.5% (33/41)	58.5% (24/41)	58.5% (24/41)	82.9% (34/41)	85.4% (35/41)	61.0% (25/41)	70.7% (29/41)	78.1% (32/41)	80.5% (33/41)
PPV	61.2% (30/49)	57.9% (33/57)	80.6% (29/36)	80.5% (33/41)	64.6% (31/48)	66% (33/50)	82.1% (32/39)	84.2 (32/38)	67.3% (33/49)	73.3% (33/45)	79.1% (34/43)	80.0% (32/40)
NPV	81.5% (22/27)	89.5% (17/19)	85.0% (34/40)	94.3% (33/35)	85.7% (24/28)	92.3% (24/26)	91.9% (34/37)	92.1% (35/38)	92.6% (25/27)	93.6% (29/31)	97.0% (32/33)	91.7% (33/36)
Accuracy	68.4% (52/76)	65.8% (50/76)	82.9% (63/76)	86.8% (66/76)	72.4% (55/76)	75.0% (57/76)	86.8% (66/76)	88.2% (67/76)	76.3% (58/76)	81.6% (62/76)	86.8% (66/76)	85.5% (65/76)

*MIP* Maximum intensity projection, *NPV* Negative predictive value, *PPV* Positive predictive value, *R1* Reader 1, *R2* Reader 2

Reading times necessary for the identification of a significant lesion did not significantly (*p* = 1.0) differ between the MIPs of the UE-MRI, VACE-MRI and ABCE-MRI protocol with 4.37 s (SD ± 3.85), 4.32 s (SD ± 2.95) and 4.70 s (SD ± 2.87).

#### Lesion conspicuity, image quality and artifact strength in MIPs

Evaluations of the lesion conspicuity are presented in Fig. 3a. For UnE-MIPs, a fair inter-rater agreement (κ = 0.39, CI: 0.25–0.53) was observed in the evaluation of the lesion conspicuity, while a moderate inter-rater agreement was observed for the VAbCE-MIPs (κ = 0.53, CI: 0.39–0.65) and AbCE-MIPs (κ = 0.58, CI: 0.45–0.71). For R1, no significant difference was observed between the three methods (*p* ≥ 0.668) in the evaluation of lesion conspicuity. For R2, a significant difference in lesion conspicuity scores distribution was noted between the UnE-MIPs and both the VAbCE-MIPs (*p* = 0.006, δ = 0.30) and ABCE-MIPs (*p* = 0.042, δ = 0.24), while no difference was observed between VAbCE-MIPs and AbCE-MIPs (*p* = 0.823).

Results of the image quality and artifact strength evaluation are presented in Fig. 3b, c. Slight inter-rater agreement on the image quality could be observed for the UnE-MIPs (κ = 0.11, CI: -0.06 to 0.30) and AbCE-MIPs (κ = 0.18, CI: 0.00–0.34), while no agreement could be observed for the VAbCE-MIPs (κ = -0.02, CI: -0.15 to 0.13). No statistically significant difference in the median of the image quality could be found between any of the methods and between the readers (*p* ≥ 0.108).

Slight inter-rater agreement was observed for the artifact strength for the UnE-MIPs (κ = 0.28, CI: 0.13–0.41), while none and fair agreement could be observed for the VAbCE-MIPs (κ = 0.02, CI: -0.10 to 0.15) and AbCE-MIPs (κ = 0.28, CI: 0.13–0.41), respectively. Significant difference (*p* ≤ 0.001) of the median could also be found for the artifact strength between the derived MIPs of the three methods and among the two readers. For both readers, a higher number of artifacts were observed for the AbCE-MIPs protocol (R1: *n* = 14; R2: *n* = 23) when compared to the VAbCE-MIPs (R1: *n* = 3, *p* = 0.002, OR: 0.18; R2: *n* = 8, *p* = 0.001, OR: 0.29). R1 also identified a higher number of artifacts in the UnE-MIPs (*n* = 23, *p* ≤ 0.001, OR: 0.09) when compared to the VAbCE-MIPs. However, for R2, no significant difference (*p* = 0.617) in the number of artifacts was observed between UnE-MIPs (*n* = 10) and VAbCE-MIPs.

Between the lesion conspicuity and artifact strength a very weak negative correlation was observed for both AbCE (ρ = -0.125) and VACE (ρ = -0.165) protocols while for the UnE protocol a weak negative correlation was observed (ρ = -0.328). Images of two example cases in which delineation of the lesion was hindered in the AbCE-MIPs are presented in Fig. 4.

#### Single-slice reading of the abbreviated protocols

Figure 2b shows the distribution of the LS-scores given by the readers for the three reading protocols using the single-slice data. Minimal inter-rater agreement in the given LS-score was observed for the UnE protocol (κ = 0.39, CI: 0.24–0.0.52) while moderate inter-rater agreement could be observed for the LS-scores given using both the VAbCE (κ = 0.66, CI: 0.53–0.0.80) and ABCE (κ = 0.64, CI: 0.49–0.76) protocols.

**Fig. 5:**
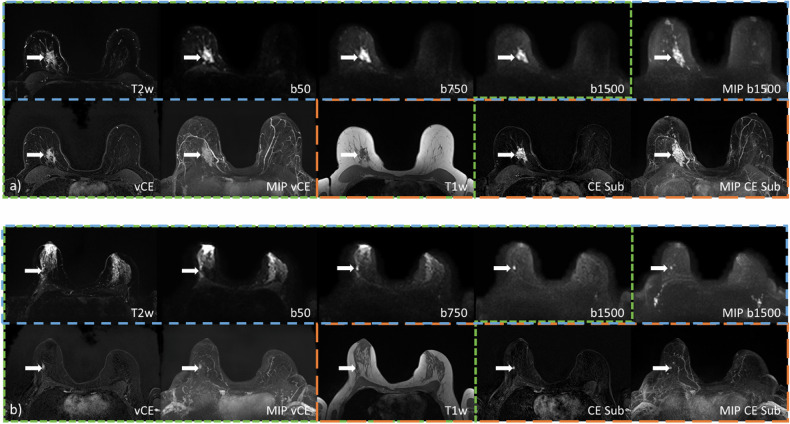
Images used during the three different reading protocols for two example cases. **a** A large nonmass enhancement (longest axis 39.9 mm) in the right breast (arrows) (histopathology invasive carcinoma no special type. **b** A small lesion (longest axis 6.6 mm) in the right breast. Sequences for the unenhanced reading protocol are surrounded by a blue border and include T2-weighted, DWI acquisitions with *b*-values of 50, 750, and 1,500 s/mm^2^ (b50, b750, b1500) and the MIP of the *b*-1,500 acquisition. Sequences which are part of the virtual abbreviated CE acquisition are marked with a green border and contain single slices of: T2-weighted, DWI with three *b*-values, T1-weighted images and the vCE image, as well as the MIP of the vCE slices. The abbreviated CE protocol includes both the slices of the T1-weighted acquisition and the CE subtraction, as well as the MIP of the CE subtraction. CE, Contrast-enhanced; DWI, Diffusion-weighted imaging; MIP, Maximum intensity projection; vCE, Virtual contrast-enhanced

Using the single-slice images, the readers achieved mean sensitivities of 88.6% (R1: 82.9%; R2: 94.3%), 91.4% (R1: 91.4%; R2: 91.4%) and 94.3% (R1: 97.1%; R2: 91.4%) for the UnE, VAbCE, and AbCE protocols, respectively. At the same time, mean specificities of 81.7% (R1: 82.9%; R2: 80.5%), 84.1% (R1: 82.9%; R2: 85.4%), and 79.3% (R1: 78.0%; R2: 80.5%) were observed for UnE, VAbCE, and AbCE protocols. In regard to the accuracies, UnE achieved a mean of 84.9% (R1: 82.9%; R2: 86.8%), VAbCE 87.5% (R1: 86.8%; R2: 88.2%), and AbCE 86.2% (R1: 86.8%; R2: 85.5%). The differences in the diagnostic accuracy between the methods and reader were non-significant (*p* ≥ 0.125). Example cases are presented in Fig. 5.

### Side-by-side comparison of image equivalence and individual lesion conspicuity of the MIP from virtual CE images (experiment 2)

**Fig. 6:**
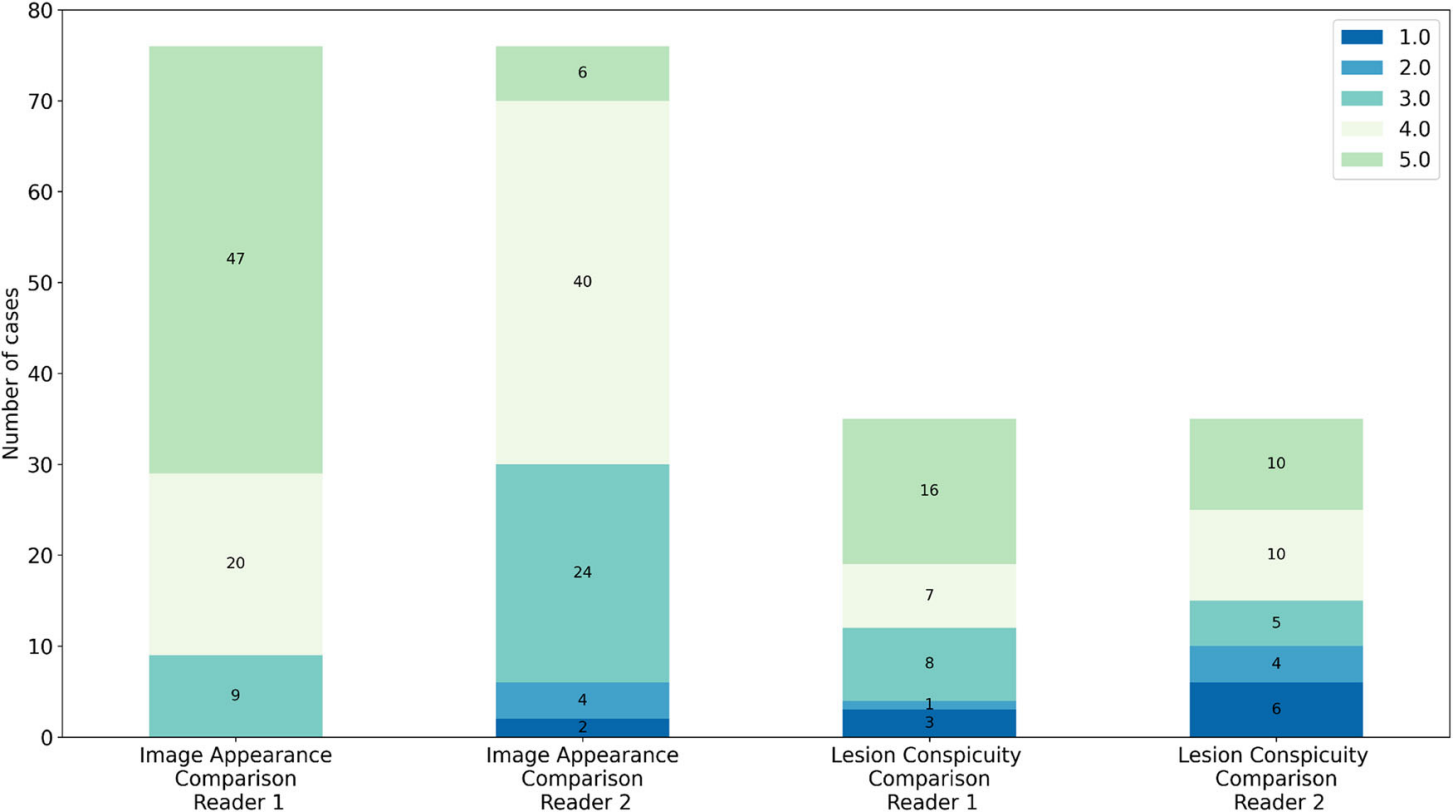
Image appearance and lesion conspicuity side-by-side comparison of VAbCE-MIPs *versus* AbCE-MIPs. Values inside bars indicate the total number of cases with the respective assigned score. For image appearance, scores correspond to: 1 = image entirely different from AbCE-MIP to 5 = image exactly equivalent to AbCE-MIP. For lesion conspicuity, scores correspond to 1 = lesion not visible to 5 = lesion enhancing equivalently to AbCE-MIP. AbCE, Abbreviated contrast-enhanced; MIP, Maximum intensity projection; VAbCE, Virtual abbreviated contrast-enhanced

Figure 6 shows results of the unblinded side-by-side comparison. Evaluation of image appearance equivalence shows for R1 median score of 5 (min-max range: 3–5) and for R2 a median of 4 (min-max range: 1–5), thus revealing not all images of the VAbCE-MIPs to show a full identical appearance as compared to the AbCE-MIPs.

**Fig. 7:**
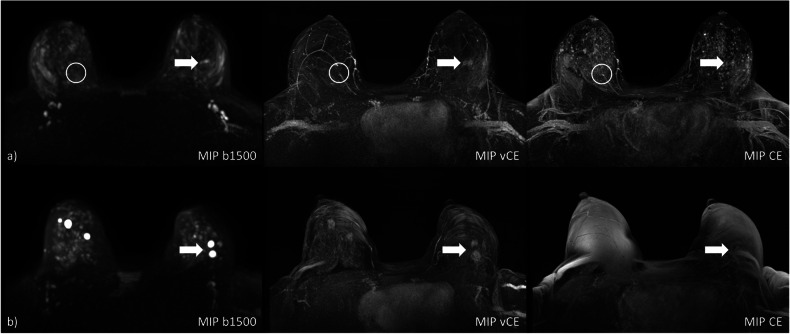
Two cases (**a**, **b**) in which the visible lesions in vCE-MIP were not corresponding to the factual lesions present in the CE acquisition. **a** A lesion was identified in the left breast of the vCE-MIP, while the DWI-MIP was called negative. The factual malignant target lesion, however, was located in the right breast (white circle), yet not enhancing after intravenous contrast injection (histopathology: mucinous carcinoma). At the same time, on the *b*-1,500-MIP, no distinct lesion was identified bilaterally. **b** The vCE-MIP shows multiple enhancing lesions in both breasts, which correspond to benign findings, *i.e*., hyperintense cysts at *b*-1,500-MIP. At the same time, the CE-MIP is not assessable due to a marked artifact. *b*-1,500, Diffusion-weighted imaging with *b* = 1,500 s/mm^2^; CE, Contrast-enhanced; MIP, Maximum intensity projection; vCE, Virtual contrast-enhanced

Evaluation of the lesion conspicuity revealed a median score of 4 for both readers given for the individual malignant lesions, with min-max scores from 1 to 5, thus revealing cases in which malignant lesions were not visually discernible in the VAbCE-MIPs. The malignant lesion conspicuity was rated as almost or fully similar as compared to the AbCE-MIPs (see Fig. 6) in 65% (23/35) and in 57% (20/35) by R1 and R2, respectively. Figure 7 shows MIPs for two cases in which lesions could be identified in the VAbCE protocol at locations not correlating with the target lesions identified on the AbCE protocol.

## Discussion

Breast MRI is increasingly evaluated with regard to the applicability to imaging protocols focused on specific fields of application, such as abbreviated protocols and reading strategies for breast cancer screening in women with dense breast tissue.

Here, we report first experience in evaluating MIPs derived from either high *b*-value DWI or a virtual CE subtraction series with subtraction MIPs from CE acquisitions. Expectedly, our study revealed the highest sensitivity to be achieved by using CE subtraction (over 94%), while both high *b*-value DWI-MIPs and the VAbCE-MIPs provided a 90–91% sensitivity. Interestingly, the addition of the artificial intelligence (AI)-generated VAbCE data to the UnE acquisition allowed for an improvement of the inter-rater agreement for the LS-score provided by the two readers.

Abbreviated CE protocols using MIPs have been investigated by different researchers with a large study being published by Kuhl et al in 2014 [7]. Using subtraction MIPs as a primary visual screening allowed to detect about 90% of cancers, which is well matched by our study data with a mean sensitivity for detecting relevant findings of 94% using the AbCE-MIPs.

DWI, including high *b*-values, is another approach investigated to avoid intravenous administration of contrast agents. Our study demonstrated a 90% sensitivity of DWI-MIPs for the detection of significant findings, which is in the range of sensitivities previously reported for high *b*-value DWI [10, 25, 31]. Interestingly, augmenting the unenhanced acquisition data by AI to derive virtual CE-MIPs did not seem to add substantial lesion detection rates, as the sensitivity increased on average by just 1.4%. When considering the lesion characterization using the single slices following the identification on MIPs, both techniques achieved similar metrics with sensitivities in the range of existing literature [9, 25, 32, 33]. Expectedly, all reading techniques of MIPs benefited from the additive reading of the single slices, improving accuracy by approximately 5%.

To our best knowledge, this publication is among the first to analyze MIPs generated from virtual CE breast MRI datasets and to compare them to both high *b*-value DWI and CE subtraction MIPs. Thus, rather only the single-slice evaluations can be compared to existing literature. As compared to the study from Mueller-Franzes et al [14], our approach generated higher scores for lesion conspicuity for the UnE data; however, these authors only included T1-weighted and T2-weighted images in the training data, not using DWI to complement training data. Chung et al [13] included DWI in their training, although not including high *b*-values as in our study. Similarly to our study, they described high image quality levels for the simulated enhancement; however, the study did not include MIPs readings and did not assess lesion conspicuity. Wang et al [20] recently performed a similar study, also not using MIPs, with a slightly smaller test cohort (*n* = 61) and using a generative adversarial network to generate virtual CE images. They did not observe a significant difference in lesion conspicuity and image quality between CE and virtual CE images. Similarly, to our work, they showed that the addition of virtual CE images to the VAbCE protocol slightly improved the sensitivity in comparison to the UnE protocol, without a significant difference. Their evaluation of VAbCE *versus* AbCE protocol showed no significant difference in both sensitivity and specificity.

Prior to the introduction of simulated CE data, UnE approaches were partially limited in the ability to use visual morphologic image assessments in the target sequence, especially for DWI [9, 10, 33], although improving DWI morphologic description [11]. Therefore, we aimed at mimicking an image contrast similar to that of CE subtraction images from UnE acquisitions. The achieved image quality and image appearance of virtual CE-MIPs were rated as mostly matching the original CE images in the side-by-side reading. Image artifacts are a common issue in MRI, with the subtraction techniques used in breast MRI being at special risk for causing artifacts due to motion-related blurring or inhomogeneities [27, 29, 34, 35]. This kind of artifact might be visible in up to 1 of 2–3 breast MRI examinations [28, 29, 34–37]. We showed that MIPs used in the VAbCE reading protocol showed a significantly lower number of visually significant artifacts when compared to the original CE-MIPs (see Fig. 3).

While virtual CE approaches are rather not aiming at universally replacing GBCA for all clinical indications of breast MRI, such techniques might facilitate accessibility and introduction of breast MRI for specific indications such as intermediate risk screening for women with dense breasts or women unable to receive gadolinium-based contrast agent.

There are several limitations to this study. First, the study cohort is relatively small (*n* = 540), possibly not reflecting all variations of breast tissue configurations and characteristics/types of breast lesions. As a result, the portion of the cohort reserved for a test set is equally limited, which in turn limits the statistical power to detect small to moderate differences between the two approaches on that test data. Further, the cohort does not reflect a screening setting, for which abbreviated breast MRI was suggested in its initial studies. Additionally, the use of data from just a single MRI scanner limits the generalizability of our method.

Of note, our study also used a complete set of acquisitions to be available for the generation of the virtual CE images. However, in clinical routine, some acquisitions might be omitted. Hence, to improve the generalizability of our method, it might be beneficial to perform training without some of the input sequences.

Certain limitations of the reader study might be the potential bias due to the lack of blinding of the information on VAbCE or AbCE data. In addition, even though the VAbCE protocol provided the reader with more comprehensive assessment possibilities in the single-slice reading than the AbCE and UnE protocols, it did not improve diagnostic assessment over the UnE protocol. Additionally, due to the limited size of our readers panel, which was comprised of just two board-certified radiologists, we did not assess the potential influence of reader experience. Future studies with larger and more specific cohorts, multiple sites and readers are needed to more comprehensively assess the potential and limitations of virtual contrast-enhanced approaches and or UE-MRI techniques.

In conclusion, UnE breast MRI using high *b*-value DWI and virtual CE provided similar accuracy for detecting malignant findings, both demonstrating a lower sensitivity when compared to original CE subtraction MIPs. Further research including the identification of potential fields of application is necessary to advance the concept of virtual CE in breast MRI.

## Supplementary information

**Additional file 1:** **Fig. S1.** U-net architecture used for the generation of a virtual dynamic contrast enhancement. The architecture uses 5 input channels, three encoder and three decoder stages. The first stage (marked with dark blue) consists of two 1 × 1 convolution layers with batch normalization and leaky rectified linear unit (LReLU) activation layer. The further two encoder stages consist each of two 3 × 3 convolution layers with batch normalization and LReLU activation layer. Between each of the encoder stages a down-sampling with a 2 × 2 convolutional layer with a stride of 2 is performed. The three decoder stages each consist of two 3 × 3 convolution layers with a batch normalization and LReLU activation layer. The upsampling is performed using a transposed 2 × 2 convolution layer with a stride of 2. The encoder and decoder satges are connected with Number of extracted features after each layer is presented below each upsampling and downsampling step.

## Data Availability

Original image data used in this work are not publicly available to preserve individuals’ privacy under the European General Data Protection Regulation. The institution handling this data is the Institute of Radiology, University Hospital Erlangen.

## Abbreviations

AbCE: Abbreviated contrast-enhanced
AI: Artificial intelligence
BI-RADS: Breast Imaging Reporting and Data System
CE: Contrast-enhanced
CI: Confidence interval
DCE: Dynamic contrast-enhanced
DWI: Diffusion-weighted imaging
LS-score: Lesion suspicion score
MIPs: Maximum intensity projections
OR: Odds ratio
R1: Reader 1
R2: Reader 2
UnE: Unenhanced
VAbCE: Virtual abbreviated contrast-enhanced
