# Supplementary material for: Virtual contrast-enhanced maximum intensity projections from high-b-value diffusion-weighted breast MRI: a feasibility study
Source: Eur Radiol Exp. 2025 Oct 8;9:100. doi: 10.1186/s41747-025-00625-7 (PMC12508357; doi:10.1186/s41747-025-00625-7)
Supplement: Supplementary file 1 — Additional file 1: Fig. S1. U-net architecture used for the generation of a virtual dynamic contrast enhancement. The architecture uses 5 input channels, three encoder and three decoder stages. The first stage (marked with dark blue) consists of two 1 × 1 convolution layers with batch normalization and leaky rectified linear unit (LReLU) activation layer. The further two encoder stages consist each of two 3 × 3 convolution layers with batch normalization and LReLU activation layer. Between each of the encoder stages a down-sampling with a 2 × 2 convolutional layer with a stride of 2 is performed. The three decoder stages each consist of two 3 × 3 convolution layers with a batch normalization and LReLU activation layer. The upsampling is performed using a transposed 2 × 2 convolution layer with a stride of 2. The encoder and decoder satges are connected with Number of extracted features after each layer is presented below each upsampling and downsampling step. [file 41747_2025_625_MOESM1_ESM.docx]

**Virtual contrast-enhanced maximum intensity projections from high-*b*-value diffusion-weighted breast MRI: a feasibility study**

**Supplemental Material**

**Methods**

**Neural network**

The used architecture is presented in Supplement Fig. S1. The architecture consisted of three encoder and three decoder stages with a bottleneck layer between the deepest encoder and decoder stage. Each stage consisted of two convolutional layers with batch normalization, and leaky rectified linear unit activation function. The down sampling was accomplished using a $2\times2$ convolution with stride 2. For the up sampling of the feature maps a transposed $2\times2$ convolution with stride 2 was used.

The network was implemented using Python software (version 3.8.10) with the use PyTorch (version 1.9.0) and PyTorch Lightning (version 1.5.5) frameworks. The network was trained using T1-weighted, T2-weighted and DWI acquisitions with three different B-values of 50, 750, and 1,500 s/mm^2^ as inputs. The input data was normalized and scaled to [0, 1]. The subtractions at the five time-points of a DCE T1-weighted acquisition were used as targets normalized scaled to [-1, 1]. During the preprocessing the volume was cropped to the volume of the DWI acquisition and the data matrix was resampled to 448 × 280 ×96.

The whole DCE series was used as the target instead of just a single post-contrast subtraction in order to improve the performance of the network by exploiting the underlying time dependency between the five post-contrast subtractions.


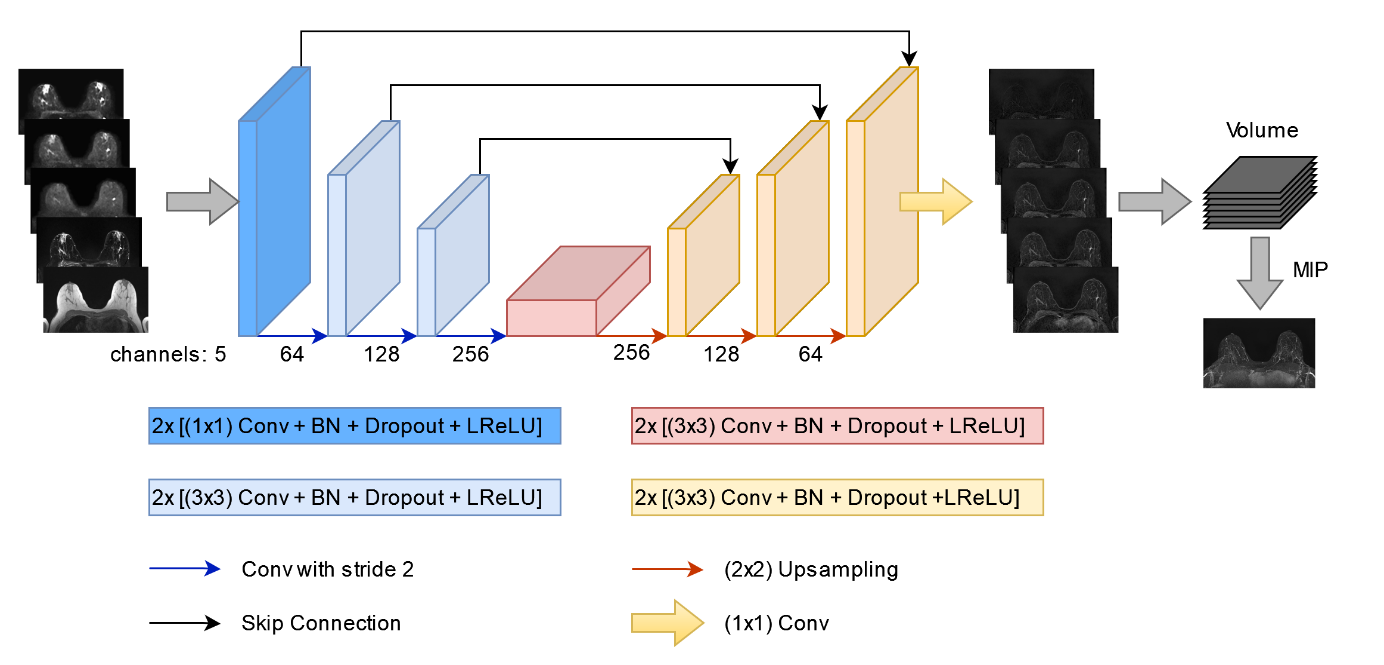


**Fig. S1** U-net architecture used for the generation of a virtual dynamic contrast enhancement. The architecture uses 5 input channels, three encoder and three decoder stages. The first stage (marked with dark blue) consists of two 1x1 convolution layers with batch normalization and leaky rectified linear unit (LReLU) activation layer. The further two encoder stages consist each of two 3 × 3 convolution layers with batch normalization and LReLU activation layer. Between each of the encoder stages a down-sampling with a 2 × 2 convolutional layer with a stride of 2 is performed. The three decoder stages each consist of two 3 × 3 convolution layers with a batch normalization and LReLU activation layer. The upsampling is performed using a transposed 2 × 2 convolution layer with a stride of 2. The encoder and decoder satges are connected with Number of extracted features after each layer is presented below each upsampling and downsampling step.

The network was trained using a loss function combining the Structural Similarity Index Measure metric and the L1 loss as suggested by Chen et al. [1; 2] However, due to the lack of segmented data the additional loss function for the lesion was omitted. During training ADAM optimizer was used with a learning rate of 10^-3^. The networks were trained for 35 epochs using a batch size of 32 on a single graphic card (NVIDIA, Quadro RTX 6000, CUDA version 11.2) with a dedicated workstation running Ubuntu version 20.04.

1 Chen C, Raymond C, Speier W et al (2023) Synthesizing mr image contrast enhancement using 3d high-resolution convnets. IEEE Trans Biomed Eng 70:401–412 <https://doi.org/10.1109/TBME.2022.3192309>

2 Schreiter H, Eberle J, Kapsner LA et al (2024) Virtual dynamic contrast enhanced breast mri using 2d u-net architectures, Deep Breast Workshop on AI and Imaging for Diagnostic and Treatment Challenges in Breast Care. Springer, pp 85–95. <https://doi.org/10.1007/978-3-031-77789-9_9>
